# The bidirectional interaction between antidepressants and the gut microbiota: are there implications for treatment response?

**DOI:** 10.1097/YIC.0000000000000533

**Published:** 2024-02-06

**Authors:** Gianluca Borgiani, Chiara Possidente, Chiara Fabbri, Vincenzo Oliva, Mirjam Bloemendaal, Alejandro Arias Vasquez, Ted G. Dinan, Eduard Vieta, Marco Menchetti, Diana De Ronchi, Alessandro Serretti, Giuseppe Fanelli

**Affiliations:** a Department of Biomedical and Neuromotor Sciences, University of Bologna, Bologna, Italy; b Departament de Medicina, Facultat de Medicina i Ciències de la Salut, Institut de Neurociències, Universitat de Barcelona (UB); c Bipolar and Depressive Disorders Unit, Hospìtal Clinic de Barcelona; d Institut d’Investigacions Biomèdiques August Pi i Sunyer (IDIBAPS), Barcelona, Spain; e Social, Genetic & Developmental Psychiatry Centre, Institute of Psychiatry, Psychology & Neuroscience, King’s College London, London, UK; f Department of Psychiatry, Radboud University Medical Center, Donders Institute for Brain, Cognition and Behaviour, Nijmegen, The Netherlands; g Department for Psychiatry, Psychosomatic Medicine and Psychotherapy, University Hospital Frankfurt-Goethe University, Frankfurt, Germany; h Department of Human Genetics, Radboud University Medical Center, Donders Institute for Brain, Cognition and Behaviour, Nijmegen, The Netherlands; i APC Microbiome Ireland; j Department of Psychiatry and Neurobehavioral Science, University College Cork, Cork, Ireland; k Centro de Investigación Biomédica en Red de Salud Mental (CIBERSAM), Instituto de Salud Carlos III, Madrid, Spain; l Department of Medicine and Surgery, Kore University of Enna, Italy; University of Ferrara, Ferrara, Italy; University of Campania, Naples, Italy; University of Bari, Bari, Italy; University of Pavia, Pavia, Italy; University of Verona, Verona, Italy; University of Florence, Florence, Italy; University of Genoa, Genoa, Italy; University Tor Vergata, Rome, Italy

**Keywords:** alpha diversity, antidepressant response, beta diversity, gut bacteria, gut-brain-axis, major depressive disorder, metabolism, personalized medicine, precision medicine, psychobiotics

## Abstract

This review synthesizes the evidence on associations between antidepressant use and gut microbiota composition and function, exploring the microbiota’s possible role in modulating antidepressant treatment outcomes. Antidepressants exert an influence on measures of gut microbial diversity. The most consistently reported differences were in β-diversity between those exposed to antidepressants and those not exposed, with longitudinal studies supporting a potential causal association. Compositional alterations in antidepressant users include an increase in the Bacteroidetes phylum, Christensenellaceae family, and Bacteroides and Clostridium genera, while a decrease was found in the Firmicutes phylum, Ruminococcaceae family, and Ruminococcus genus. In addition, antidepressants attenuate gut microbial differences between depressed and healthy individuals, modulate microbial serotonin transport, and influence microbiota’s metabolic functions. These include lyxose degradation, peptidoglycan maturation, membrane transport, and methylerythritol phosphate pathways, alongside gamma-aminobutyric acid metabolism. Importantly, baseline increased α-diversity and abundance of the Roseburia and Faecalibacterium genera, in the Firmicutes phylum, are associated with antidepressant response, emerging as promising biomarkers. This review highlights the potential for gut microbiota as a predictor of treatment response and emphasizes the need for further research to elucidate the mechanisms underlying antidepressant-microbiota interactions. More homogeneous studies and standardized techniques are required to confirm these initial findings.

**See related paper on page 46**

## Introduction

The human enteric tract is estimated to be populated by 3.9 × 10^13^ microorganisms, with a 1:1 ratio of bacteria to human cells, and incorporates 100 times the genes in the human genome (Dinan and Cryan, 2015; Sender *et al.*, 2016). Advances in DNA sequencing and bioinformatic analysis have enabled a better understanding of gut microbiota composition and function. A growing body of research highlights the significant role of the gut microbiota in brain functioning and psychological well-being (Dinan and Cryan, 2020). The interaction between the gut microbiota and the brain occurs through various pathways, including the immune system, the vagus nerve and enteric nervous system, enteroendocrine signaling, and the hypothalamic-pituitary-adrenal axis (Dinan and Cryan, 2017). This complex bidirectional communication is commonly referred to as the brain-gut-microbiota axis (Cryan *et al.*, 2019). Microbial metabolites such as short-chain fatty acids, branched-chain amino acids, and peptidoglycans are key components in this communication system (Cryan *et al.*, 2019).

Several psychiatric disorders, including anxiety disorders, anorexia nervosa, obsessive-compulsive disorder (OCD), and major depressive disorder (MDD) have been linked to changes in gut microbiota composition (Clapp *et al.*, 2017; Slyepchenko *et al.*, 2017; Valles-Colomer *et al.*, 2019b; Turna *et al.*, 2020). The observed changes exhibit a transdiagnostic pattern, characterized by a reduction in anti-inflammatory butyrate-producing bacteria and an increase in pro-inflammatory bacteria (Nikolova *et al.*, 2021; McGuinness *et al.*, 2022). This imbalance leads to the development of a chronic inflammatory state and, ultimately, heightened reactivity to stress and an increased vulnerability to stress-related psychiatric disorders (Foster *et al.*, 2017). Additionally, the gut microbiota can influence the synthesis and metabolism of neurotransmitters, thereby affecting brain function through modulation of various neurotransmission systems involved in the development and progression of neuropsychiatric disorders, such as the noradrenergic, serotoninergic, dopaminergic, glutamatergic, and gamma-aminobutyric acid (GABA)-ergic systems (Socała *et al.*, 2021). Serotonin, in particular, is the predominant neurotransmitter in the gut, with up to 95% of total body serotonin secreted by gut mucosal enterochromaffin cells and enteric neurons (Guzel and Mirowska-Guzel, 2022). Bacterial enzymes can influence serotonin levels in the brain by modulating the metabolic pathway of tryptophan, a serotonin precursor, and consequently the entire serotonergic system (O’Mahony *et al.*, 2015; Socała *et al.*, 2021).

Antidepressants work mainly by increasing the bioavailability of serotonin and other neurotransmitters such as noradrenaline and dopamine, and are used in a broad range of psychiatric disorders, including anxiety disorders, OCD, and MDD, in addition to several other off-label uses (Serretti, 2022b; Sheffler and Abdijadid, 2022). Antidepressants influence the gut microbiota both directly through proven antimicrobial activity and indirectly by altering the environment for microbial growth (Maier *et al.*, 2018; Ait Chait *et al.*, 2020). The impact of antidepressants on gut microbial balance may also contribute to the gastrointestinal side effects commonly associated with these medications (Oliva *et al.*, 2021). On the other hand, bacteria have the potential to affect drug metabolism and modulate the efficacy and toxicity of various medications, although limited knowledge exists in this area (Cussotto *et al.*, 2021). This could help explain unresolved issues about antidepressants, including the inter-individual differences in efficacy and side effects (Cipriani *et al.*, 2018), the occurrence of tachyphylaxis (Kinrys *et al.*, 2019), and treatment resistance (De Carlo *et al.*, 2016). Tailoring prescriptions based on individual characteristics, potentially including the gut microbiome profile, holds promise for improving treatment outcomes and preventing chronicity (Serretti, 2018; McGuinness *et al.*, 2022).

Therefore, we reviewed previous preclinical and clinical evidence to determine (1) whether the composition and diversity indices of gut microbiota may be cross-sectionally associated with antidepressant use, as well as (2) whether antidepressant treatment may longitudinally result in a change in the microbiota compositional profile. Additionally, we investigated (3) the potential effects of antidepressants on the gut microbiota functional profile (i.e., . the gut microbiome metabolic pathways). Finally, we examined (4) whether any microbiota feature could exert an influence on antidepressant efficacy or serve as a biomarker of treatment outcomes.

## Materials and methods

### Search strategy

A literature search was conducted on PubMed/MEDLINE from inception up to November 2023. The search query included various terms related to microbiota and gut, as well as the names of commonly used antidepressant classes [i.e., selective serotonin reuptake inhibitors (SSRIs), norepinephrine reuptake inhibitors (NRIs), serotonin-NRIs (SNRIs), monoamine oxidase inhibitors (MAOIs), serotonin antagonists and reuptake inhibitors (SARIs), and tricyclic antidepressants (TCAs)] and the names of single drugs (including amitriptyline, bupropion, citalopram, clomipramine, desvenlafaxine, duloxetine, fluoxetine, fluvoxamine, escitalopram, ketamine, mirtazapine, nortriptyline, paroxetine, sertraline, trazodone, venlafaxine, and vortioxetine). The complete search query can be found in the Appendix of this article. The references of eligible studies were manually scrutinized to identify any possible studies that could have been overlooked by the initial search string. Nonetheless, it should be noted that this was intended to be a narrative and not a systematic review. This decision was made due to the broad variety of studies available in the literature, encompassing diverse designs and methodological approaches, making it challenging to assess them in a systematic manner.

Observational and experimental studies in human and animal models were considered potentially eligible. To be included in this review, the studies had to investigate either: (1) the relationship between the use of antidepressants and the composition and diversity of the gut microbiota in a cross-sectional design; (2) the longitudinal association between the use of antidepressants and the composition and diversity of the gut microbiota; (3) the relationship between antidepressant use and the metabolic activity of gut microbiota; (4) the association between gut microbiota and antidepressant treatment outcomes. Studies were excluded if they: (1) were case reports, case series, letters (if they did not report new original findings), book chapters, or review articles (except for meta-analyses); (2) were not written in English; (3) conducted on animals other than mammals; (4) considered antidepressant treatment only as a confounding variable in the analyses.

Two authors (G.B. and C.P.) performed an independent search of the articles and selected the relevant ones. In cases of disagreement during the study selection step, a decision was reached through open discussion and, in case of persistent disagreement, with the involvement of a supervisor (G.F.).

The following data were independently extracted from each study, when available, using a pre-designed form which included: (1) year of publication; (2) type of study design; (3) animal or human study; (4) sample size; (4) diagnosis (if humans) or animal model type; (5) females/males ratio; (6) mean age if humans; (7) main predictor in the analyses; (8) covariates/confoundings; (9) outcomes; (10) studied antidepressant(s); (11) time of exposure (days/months/years); (12) drug(s) dose(s); (13) studied microbe(s) and measurement technique/biospecimen; (14) main findings (Table 1).

**Table 1:** Animal and human studies investigating the bidirectional relationship between antidepressant use and gut microbiota

Reference	Study design	Study subjects, sample size (N), and sample characteristics	Main outcome(s)	Studied antidepressant(s), dose(s), and time of exposure (if available)	Measurement technique	Main findings
Preclinical studies						
(An *et al.*, 2020)	Cross-sectional	C57BL/6J mice, CUMS HCs + vehicle N = 5 CUMS + vehicle N = 5 CUMS + NMDEA N = 5	Bacterial taxonomic composition, α-diversity (Observed OTUs, Shannon, ACE, Simpson), β-diversity (unweighted UniFrac)	NMDEA (analogue of agomelatine) 30 mg/kg/day 28 days	Amplification and sequencing of 16S rRNA genes (V3-V4 regions)	CUMS disrupted the richness of the gut bacterial community. NMDEA administration made the gut microbiome more similar to that of HCs.
(Aydin *et al.*, 2021)	Cross-sectional	Sprague–Dawley rats Normoglycemic group (N = 12, reboxetine = 6), diabetic group (N = 12, reboxetine = 6)	Bacterial taxonomic composition, α-diversity (observed OTUs, Shannon index, phylogenetic diversity), β-diversity (unweighted and weighted UniFrac)	Reboxetine 8 mg/kg/day 2 weeks	Amplification and sequencing of 16S rRNA genes (V3-V4 regions)	Reboxetine use was associated with significant changes in specific taxa and led to an altered gut microbiome with increased inflammatory capacity.
(Cussotto *et al.*, 2019)	Cross-sectional	Sprague–Dawley rats Controls N = 8 Escitalopram N = 8 Venlafaxine N = 8 Fluoxetine N = 8 Lithium N = 8 Valproate N = 8 Aripiprazole N = 8	Bacterial taxonomic composition, α-diversity (Shannon, Chao1), β-diversity (Bray Curtis)	Escitalopram: 6.38 mg/kg/day venlafaxine: 20 mg/kg/day fluoxetine: 10 mg/kg/day 28 days	Amplification and sequencing of 16S rRNA genes (V3-V4 regions)	Microbial richness and diversity in antidepressant-administered animals did not significantly differ from those of controls. Fluoxetine administration produced a decrease in the phylum Deferribacteres and in the genera Prevotella and Succinivibrio.
(Dangoor *et al.*, 2021)	Longitudinal	Wistar rats, Corticosterone-induced stress HCs N = 30 Corticosterone-induced stress N = 40	Microbiota composition shift pre-post treatment: (Beta diversity: weighted and unweighted UniFrac)	Citalopram 10 mg/kg/day 2 weeks	Amplification and sequencing of 16S rRNA genes	Both oxytocin administration and corticosterone stress induction led to significant changes in bacterial composition. No such change was observed with citalopram.
(Dethloff *et al.*, 2020)	Longitudinal	DBA/2J mice, depressed mouse strain Paroxetine N = 17 vehicle N = 17	Bacterial taxonomic composition α-diversity (Faith’s Phylogenetic Diversity) β-diversity (unweighted and weighted UniFrac) Bile acid levels	Paroxetine 10 mg/kg/day 2 weeks	Amplification and sequencing of 16S rRNA genes	There were several primary and secondary bile acid level and microbiota α-diversity differences between paroxetine- and vehicle-treated mice
(Diviccaro *et al.*, 2022)	Longitudinal	Sprague–Dawley rats N = N/A	Bacterial taxonomic composition α-diversity (Evenness, Shannon, Faith, and Observed Features metrics) β-diversity (Bray Curtis, Unweighted and Weighted Unifrac)	Paroxetine 10 mg/kg/day 2 weeks	Amplification and sequencing of 16S rRNA genes (V4 regions)	Paroxetine did not affect α-diversity but altered β-diversity. Taxa belonging to the Firmicutes phylum were significantly altered with a significant increase in the Enterococcaceae family, belonging to the Bacilli class, and several variations related to the Clostridia class.
(Dong *et al.*, 2023)	Longitudinal	Sprague–Dawley rats, CUMS HCs N = 10 HCs + fluoxetine N = 10 CUMS N = 10 CUMS + fluoxetine N = 10	Bacterial taxonomic composition α-diversity (Shannon, Simpson, Chao 1, ACE) β-diversity (Unweighted Unifrac)	Fluoxetine 1 mg/kg/day 4 weeks	Amplification and sequencing of 16S rRNA genes (V4 region)	Fluoxetine administration did not restore CUMS induced alterations. Possible fluoxetine under dosage.
(Duan *et al.*, 2021)	Longitudinal	C57BL/6 mice, CUMS HCs N = 8 CUMS+vehicle N = 7 Responders N = 7 Non-responders N = 9	Bacterial taxonomic composition α-diversity (Shannon, ACE, Chao1, Simpson) β-diversity (unweighted UniFrac) Serum metabolic signatures	Escitalopram 10 mg/kg/day 4 weeks	Amplification and sequencing of 16S rRNA genes (V3-V4 regions)	Increased α-diversity of the gut microbiome in the escitalopram treatment group. The responder group was mainly characterized by increased levels of genus Prevotellaceae UCG-003, and the non-responder group by depleted families Ruminococcaceae and Lactobacillaceae
(Fung *et al.*, 2019)	Longitudinal	C57Bl/6 mice N = 14	Bacterial taxonomic composition α-diversity (Observed OTUs)	Fluoxetine 10 mg/kg/day 7 days	Amplification and sequencing of 16S rRNA genes (V4 regions)	Fluoxetine administration led to a decreased abundance of Turicibacter and Clostridiaceae, with no significant differences in α-diversity.
(Getachew *et al.*, 2018)	Cross-sectional	Wistar rats Saline N = 5 Ketamine N = 5	Bacterial taxonomic composition α-diversity (Observed species, Shannon)	Ketamine 2.5 mg/kg/day 7 days	Amplification and sequencing of 16S rRNA genes (V3-V4 regions)	Ketamine reduced levels of opportunistic pathogens like Ruminococcus and Mucispirillum and elevated those of the beneficial genera Lactobacillus, Sarcina, and Turicibacter.
(Huang *et al.*, 2019)	Cross-sectional	C57BL/6 mice, inflammation model of depression (LPS) HCs N = 8 LPS+ saline N = 8 LPS+ ketamine N = 8	Bacterial taxonomic composition α-diversity (Shannon, Simpson, Chao1) β-diversity (Bray Curtis)	Ketamine hydrochloride 10 mg/kg single intraperitoneal administration	Amplification and sequencing of 16S rRNA genes (V4-V5 regions)	The phylum Actinobacteria, the class Coriobacteriia, the order Clostridiales, the family Prevotellaceae, and the genus Alloprevotella independently correlated with treatment outcomes and might be potential biomarkers for ketamine’s antidepressant efficacy.
(Kim *et al.*, 2021)	Cross-sectional	C57BL/6 N mice, immobilization stress, Escherichia Coli stress HCs: N = 6 immobilization stress N = 6 immobilization stress + Buspirone orally N = 6 immobilization stress + Buspirone intraperitoneally N = 6 HCs N = 6 Escherichia Coli stress N = 6 Escherichia Coli stress + Buspirone orally N = 6 Escherichia Coli stress + Buspirone intraperitoneally N = 6	Bacterial taxonomic composition α-diversity (Observed OTUs) β-diversity (generalized UniFrac analysis)	Buspirone - orally: 5 mg/kg/day, - intraperitoneally injected: 1 mg/kg/day 5 days	Amplification and sequencing of 16S rRNA genes (V4 regions)	Buspirone partially minimized differences in gut microbiota between the stressed group and HCs (including β-diversity).
(Lukić *et al.*, 2019)	Cross-sectional	BALB/c OlaHsd mice, depressed mouse strain HCs N = 9 Fluoxetine N = 11 Escitalopram N = 12 Venlafaxine N = 12 Duloxetine N = 11 Desipramine N = 12	Bacterial taxonomic composition α-diversity (Faith’s phylogenetic diversity, Chao1, Gini coefficient) β-diversity (unweighted and weighted UniFrac)	Fluoxetine: 10 mg/kg/day escitalopram: 10 mg/kg/day venlafaxine: 10 mg/kg/day duloxetine: 10 mg/kg/day desipramine: 20 mg/kg/da 3 weeks	Amplification and sequencing of 16S rRNA genes (V4 regions)	All the drugs except desipramine reduced the richness of the gut microbiome but did not affect its evenness, while simultaneously β-diversity of antidepressant groups was found to be higher than that of control samples. Supplementation with Ruminococcus Flavefaciens was able to abolish the antidepressant effects of duloxetine
(Lyte, Daniels and Schmitz-Esser, 2019)	Longitudinal	CF-1 mice HCs: N = 10 Fluoxetine N = 10	Bacterial taxonomic composition α-diversity (observed species, Shannon, ACE, Chao1, Simpson)	Fluoxetine 20 mg/kg/day 29 days	Amplification and sequencing of 16S rRNA genes (V4 regions)	Fluoxetine induced a significant, time-dependent decrease in the abundance of beneficial Lactobacillus johnsonii spp.
(McVey Neufeld *et al.*, 2019)	Longitudinal	BALB/c mice, depressed mouse strain N = 5	Bacterial taxonomic composition α-diversity (Shannon, Simpson) β-diversity (Bray Curtis)	Sertraline 6 mg/kg/day 2 weeks	Amplification and sequencing of 16S rRNA genes (V3 regions)	While sertraline treatment did not alter the overall microbial profile, there was a significant decrease in α-diversity over the treatment period that was not observed in controls.
(Qu *et al.*, 2017)	Cross-sectional	C57BL/6 mice, social defeat stress model of depression HCs N = 6 Saline N = 6 (R)-ketamine N = 6 Lanicemine N = 6	Bacterial taxonomic composition β-diversity (Bray Curtis)	(R)-ketamine 10 mg/kg single intraperitoneal injection	Amplification and sequencing of 16S rRNA genes (V4 regions)	(R)-ketamine exerted its antidepressant effect by increasing decreased levels of the order Bacteroidales, Clostridiales, and family Mogibacteriaceae, and decreasing the levels of the genus Clostridium and family Ruminococcaceae.
(Ramsteijn *et al.*, 2020)	Longitudinal	Wistar rats, Early life stress HCs + Vehicle N = 11 Stressed + Vehicle N = 8 HCs + Fluoxetine N = 8 Stressed + Fluoxetine N = 9 Early life stress	Bacterial taxonomic composition α-diversity (Shannon) β-diversity (weighted UniFrac) Inferred metabolic pathways (PICRUSt)	Fluoxetine 10 mg/kg/day during pregnancy and lactation	Amplification and sequencing of 16S rRNA genes (V4 regions)	Fluoxetine treatment modulated key aspects of maternal microbial community dynamics and metabolite output, mainly in early-life stressed females.
(Shen *et al.*, 2023)	Longitudinal	C57/6 mice, CUMS HCs N = 10 CUMS + vehicle N = 10 CUMS + venlafaxine N = 10	Bacterial taxonomic composition α-diversity (Shannon, Simpson, Chao and Ace)	Venlafaxine 12.5 mg/kg/day 2 weeks	Amplification and sequencing of 16S rRNA genes (V4 regions)	Venlafaxine made the gut microbiome more similar to that of HCs restoring CUMS alterations.
(Sun *et al.*, 2019)	Cross-sectional	C57/6 mice, CUMS HCs N = 10 CUMS + vehicle N = 10 CUMS+ fluoxetine N = 10	Bacterial taxonomic composition α-diversity (Shannon) β-diversity (Bray Curtis)	Fluoxetine 12 mg/kg/day 3 weeks	Amplification and sequencing of 16S rRNA genes	Fluoxetine made the gut microbiome more similar to that of HCs
(Vuong *et al.*, 2021)	Longitudinal	C57BL/6 J mice Saline N = 6 Fluoxetine N = 6 Antibiotics + saline N = 6 antibiotics + fluoxetine N = 6	Bacterial taxonomic composition α-diversity (Shannon) β-diversity (weighted UniFrac)	Fluoxetine 10 mg/kg/day 8 days	Amplification and sequencing of 16S rRNA genes (V4 regions)	Maternal fluoxetine treatment had no overt effect on the composition of the maternal gut microbiota.
(Wang *et al.*, 2021)	Longitudinal	C57BL/6 mice, inflammation model of depression (LPS-induced) N = N/A	Bacterial taxonomic composition α-diversity (Shannon, Simpson) β-diversity (Weighted UniFrac, Binary Jaccard)	(S)-norketamine, (R)-norketamine 10 mg/kg single intraperitoneal injection	Amplification and sequencing of 16S rRNA genes (V3-V5 regions)	(S)-nor-ketamine exerted potent antidepressant-like effects in lipopolysaccharide-induced mice, as it made the gut microbiota composition of stressed mice more similar to that of HCs.
(Yang *et al.*, 2017)	Cross-sectional	C57BL/6 mice, social defeat stress model of depression HCs N = 6 Saline N = 6 (R)-ketamine N = 6 (S)-ketamine N = 6	Bacterial taxonomic composition β-diversity (Bray Curtis, Euclidean dissimilarity)	(R)-ketamine, (S)-ketamine 10 mg/kg single intraperitoneal administration	Amplification and sequencing of 16S rRNA genes (V4 regions)	Gut microbiota partially mediated the antidepressant actions of (R)-ketamine, mainly through the elevation of decreased levels of the Mollicutes class and Butyricimonas genus.
(Zhang *et al.*, 2021)	Longitudinal	Sprague–Dawley rats, CUMS HCs N = 12 CUMS N = 36 (Fluoxetine N = 7, Amitriptyline N = 6)	Bacterial taxonomic composition α-diversity (Shannon, Simpson, Chao1) β-diversity (unweighted and weighted UniFrac) Inferred metabolic pathways	Fluoxetine: 12 mg/kg/day amitriptyline: 25 mg/kg/day 8 days	Amplification and sequencing of 16S rRNA genes	Administration of amitriptyline and fluoxetine reversed part of the gut microbiota profile and functions altered by CUMS.
Clinical studies						
(Bharwani *et al.*, 2020)	Longitudinal	MDD (N = 15) F/M:12/3 Mean age: 36.9 ± 12.9	Bacterial taxonomic composition α-diversity (Faith’s phylogenetic diversity) β-diversity (Bray-Curtis)	Citalopram, escitalopram 6 months	Amplification and sequencing of 16S rRNA genes	Remitters showed higher baseline α-diversity, which remains evident following 6 months of treatment. Bacteria from the Clostridiales order were elevated only in remitters after 6 months of treatment vs baseline. 35 OTUs were significantly different between remitters and nonremitters at 3-month follow-up, and 42 OTUs were different at 6-month follow-up.
(Dong *et al.*, 2022)	Longitudinal	MDD N = 63 HCs N = 30 F/M: 63/30 Mean age: 28.34 (MDD), 29.23 (HCs)	Bacterial taxonomic composition α-diversity (Chao1, Shannon, Simpson) β-diversity (Bray Curtis) Metabolic pathways (GC-MS)	Citalopram, escitalopram, paroxetine, venlafaxine 42.33 ± 11.88 mg/day (Fluoxetine-equivalent dose) 8 weeks	Amplification and sequencing of 16S rRNA genes (V3-V4 regions)	The abundance of the phylum Actinobacteria, families Christensenellaceae and Eggerthellaceae, genera Adlercreutzia, and Christensenellaceae R-7 group was significantly lower at baseline in responders than in non-responders.
(Falony *et al.*, 2016)	Cross-sectional	General population N = 2241 Mean age: 50,9 ± 13,6	Bacterial taxonomic composition α-diversity (observed genera, Pielou’s index, Fisher’s α) β-diversity (Bray-Curtis, Jensen-Shannon)	Venlafaxine	Amplification and sequencing of 16S rRNA genes (V4 regions)	There was a significant correlation between venlafaxine use and gut microbiome composition, with an increase in the genus Clostridium IV.
(Fontana *et al.*, 2020)	Cross-sectional	MDD HCs N = 20 N = 8 treatment resistant (TR) N = 19 responders (R) N = 7 untreated (U) F/M:31/23 Mean age: 58.8(TR), 53.7 (R), 57.0 (U), 37.7 (HCs)	Bacterial taxonomic composition	SSRIs/SNRIs/TCAs/ Serotonin modulator TR = 44.0 months (median) R = 24.0 months (median)	Amplification and sequencing of 16S rRNA genes (V3-V4 regions)	The gut microbiota of TR patients significantly differed from responders for the families of Paenibacillaceae and Flavobacteriaceaea, for the genus Fenollaria, and the species Flintibacter butyricus, Christensenella timonensis, and Eisenbergiella massiliensis, among others. The phyla Proteobacteria, Tenericutes and the family Peptostreptococcaceae were more abundant in TR patients, whereas the phylum Actinobacteria was enriched in responders.
(Gao *et al.*, 2023)	Cross-sectional, Longitudinal	MDD N = 62 N = 25 treatment resistant (TR) N = 37 responders (R) HCs N = 41 F/M: 53/41 Mean age: 24.4 (TR), 22.67 (R), 23.34 (HCs)	Bacterial taxonomic composition	SSRIs 8 weeks	Amplification and sequencing of 16S rRNA genes (V3-V4 regions)	The relative abundance of Blautia, Coprococcus, and Bifidobacterium was elevated in the responsive patients with MDD
(Imhann *et al.*, 2016)	Cross-sectional	General population N = 1174 F/M: 688/486 Mean age: 48,36 ± 13,58	Bacterial taxonomic composition α-diversity (observed species, Shannon) β-diversity (Bray-Curtis)	SSRIs, SNRIs, Mirtazapine, Tricyclics	Amplification and sequencing of 16S rRNA genes (V4 regions)	The increase of two taxa (belonging to Bacteroidetes phylum) was significantly associated with antidepressant use, but not after correction for PPI use.
(Jackson *et al.*, 2018)	Cross-sectional	General population N = 2737 F/M: 2435/302 Mean age: 60 ± 12	Bacterial taxonomic composition α-diversity (Shannon index, phylogenetic diversity, raw OTUs counts) β-diversity (weighted and unweighted UniFrac)	SSRIs, TCAs	Amplification and sequencing of 16S rRNA genes (V4 regions)	Significant associations between antidepressant medications and gut microbiota markers were found. SSRIs were negatively associated with Turicibacteraceae abundance.
(Jiang *et al.*, 2015)	Cross-sectional	MDD N = 29 Active-MDD N = 17 responders HCs N = 30 F/M: 34/42 Mean age: 25,3 (A-MDD), 27,1 (R), 26,8 (HCs)	Bacterial taxonomic composition α-diversity (observed OTUs, Chao1, ACE, Shannon, Simpson) β-diversity (unweighted UniFrac) Serum cytokines	SSRIs, SNRIs 4 weeks	Amplification and sequencing of 16S rRNA genes (V1-V3 regions)	Increased α-diversity was found in the active-MDD vs. the HC group but not in the responders vs. the HC group. Bacteroidetes, Proteobacteria, and Actinobacteria strongly increased in level, whereas Firmicutes were significantly reduced in the A-MDD and R-MDD groups compared to the HC group.
(Lin *et al.*, 2024)	Cross-sectional	Mood Disorders N = 271 F/M: 172/89 Mean age: 44.31 (SSRI/SNRI), 43.25 (Other AD), 43.41 (No AD)	Bacterial taxonomic composition α-diversity (Faith’s, Shannon) β-diversity (unweighted UniFrac)	SSRIs, SNRIs	Amplification and sequencing of 16S rRNA genes (V3-V4 regions)	SSRIs and SNRIs showed reduced alpha diversity.
(Kurokawa *et al.*, 2021)	Cross-sectional, Longitudinal	MDD N = 16 (non-responders) N = 11 (responders) N = 6 (stable remitters) F/M:17/16 Mean age: 49.1 (responders), 53 (non-responders), 56.3 (stable remitters)	Bacterial taxonomic composition α-diversity (Chao 1, Shannon, Phylogenetic diversity) β-diversity (unweighted and weighted UniFrac) Inferred Metabolic pathways (PICRUSt)	Mixed antidepressants	Amplification and sequencing of 16S rRNA genes (V1-V2 regions)	α-diversity was lower in non-responders compared to responders during the treatment course, while no significant differences in baseline α-diversity indices were detected. Non-responders presented increased estimated glutamate synthesis microbiota functions compared to responders and stable remitters.
(Lee *et al.*, 2022)	Pilot randomized controlled trial	MDD Placebo N = 10 Levomilnacipram N = 7 F/M:7/10 Mean age: 73 (placebo), 70 (levomilnacipram)	Response to antidepressant treatment (Depressive symptom severity at baseline and follow-up assessed using the HDRS_24_)	Levomilnacipram 120 mg/day 12 weeks	Amplification and sequencing of 16S rRNA genes (V4 regions)	Baseline enrichment of Faecalibacterium, Agathobacter, and Roseburia was associated with remission.
(Liśkiewicz *et al.*, 2019)	Longitudinal	MDD N = 17 F/M: 9/8 Mean age: 42.5 ± 13.9 years	Bacterial taxonomic composition α-diversity (Observed OTUs, Chao1, Shannon, inverse Simpson) β-diversity (Bray-Curtis)	Escitalopram 5–20 mg/day 6 weeks	Amplification and sequencing of 16S rRNA genes (V4 regions)	Escitalopram treatment led to increased α-diversity, although no significant differences in taxa abundance were found at different time points.
(Liśkiewicz *et al.*, 2021)	Longitudinal	MDD N = 16 F/M: 8/8 Mean age: 42.9 ± 14.3 years	Severity of depressive symptoms (HDRS_24_), Therapeutic response (early improvement, treatment response, remission from depression, dHDRS_24_)	Escitalopram 5–20 mg/day 6 weeks	Amplification and sequencing of 16S rRNA genes (V4 regions)	The baseline abundance of different taxa was associated with the severity of depressive symptoms, but no changes in the microbiota composition were detected after clinical improvement.
(Madan *et al*., 2020)	Longitudinal	N = 111 (SUD, MDD, Bipolar Spectrum, Anxiety Spectrum, Psychotic Spectrum, Personality Disorders) F/M: 60/51 Mean age: 35.7 ± 13.8	Suicide, trauma, depression and anxiety severity, treatment response	Mixed antidepressants	Amplification and sequencing of 16S rRNA genes (V4 regions), whole genome shotgun sequencing	Gut microbiota richness and α-diversity early in the course of hospitalization were significant predictors of depression remission at discharge.
(Minichino *et al.*, 2023)	Meta-analysis of longitudinal and cross-sectional studies (only longitudinal studies were meta-analyzed for antidepressants)	19 studies included (8 on subjects treated with antidepressants)	Bacterial taxonomic composition α-diversity β-diversity Inferred metabolic pathways Association between gut microbiome and measures of treatment response and tolerability	Escitalopram, vortioxetine, mixed antidepressants	-	Significant changes were found in β-diversity but not α-diversity metrics following treatment with antidepressants. Antidepressant treatment was consistently associated with an increased abundance of the Christensenellaceae family.
(Rogers and Aronoff, 2016)	Cross-sectional	Community residents N = 1135 F/M: 103/48 Mean age: 52	Bacterial taxonomic composition α-diversity (Shannon, inverse Simpson)	Mixed antidepressants (cited citalopram)	Amplification and sequencing of 16S rRNA genes (V3-V5 regions)	Citalopram was significantly associated with an increased abundance of a specific OTU of the Enterobacteriaceae family. Moreover, adding antidepressants to NSAIDs led to changes in gut microbiota composition.
(Shen *et al.*, 2021)	Longitudinal	MDD N = 30 HCs N = 30 F/M:28/32 Mean age: 44.83 (MDD), 43.97(HCs)	Bacterial taxonomic composition α-diversity (ACE, Chao1, Shannon, Simpson) β-diversity (Binary Jaccard) Inferred Metabolic pathways (PICRUSt)	Escitalopram 16.33 ± 3.46 mg/day 4–6 weeks	Amplification and sequencing of 16S rRNA genes (V3-V4 regions)	The intestinal flora of depressed patients tended back to normal under escitalopram treatment.
(Stanislawski *et al.*, 2021)	Cross-sectional	N = 331, BD1/2 (17%), anxiety disorders (38%), PTSD (49.8%) F/M: 56/275 Mean age: 47.6	Bacterial taxonomic composition α-diversity (Observed species, Shannon diversity, and Faith’s phylogenetic diversity) β-diversity (unweighted and weighted UniFrac, Jaccard, and Bray-Curtis) Inferred Metabolic pathways (PICRUSt)	Atypical antidepressants (trazodone, bupropion), SSRIs, or SNRIs	Amplification and sequencing of 16S rRNA genes	SNRI use significantly correlated with unweighted UniFrac, implying that it is associated with significant shifts in some taxa. Furthermore, the serotonin antagonist and reuptake inhibitor (SARI) trazodone showed an inverse relationship with α-diversity.
(Thapa *et al.*, 2021)	Cross-sectional	MDD N = 110 HCs N = 27 PCs N = 23 F/M:92/98 Mean age: 19.5 (MDD), 26.3 (HCs), 19.1 (PCs)	Bacterial taxonomic composition α-diversity (Observed OTUs, Chao1, ACE, Phylogenetic diversity, Shannon) β-diversity (unweighted and weighted UniFrac, Bray Curtis)	SSRIs	Amplification and sequencing of 16S rRNA genes (V4 regions)	Using SSRIs was not associated with differential bacterial composition or diversity.
(Ticinesi *et al.*, 2017)	Cross-sectional	N = 76, Elderly patients (chronic comorbidities) HCs N = 25 F/M: 37/39 Mean age: 83.3 ± 7.5	Bacterial taxonomic composition α-diversity (Chao1, Shannon) β-diversity (unweighted UniFrac)	Mixed antidepressants	Amplification and sequencing of 16S rRNA genes (V3 regions)	Antidepressant use was positively correlated with several taxa (Asteroleplasma, Helicobacter, Marinilactibacillus, unclassified members of the Bacilli class, and Succinivibrionacae family)
(Tomizawa *et al.*, 2021)	Longitudinal	MDD N = 24 Persistent depressive disorder N = 8 Panic disorder N = 1 General anxiety disorder N = 6 Social anxiety disorder N = 1 F/M:23/17 Mean age: 54.4 ± 19.00	α-diversity (Chao1, Shannon, Faith’s phylogenetic diversity) β-diversity (unweighted and weighted UniFrac) Inferred Metabolic pathways (PICRUSt, GBM)	Amitriptyline, amoxapine, sertraline, paroxetine, escitalopram, duloxetine, venlafaxine, milnacipran, mirtazapine 142 ± 364.50 days	Amplification and sequencing of 16S rRNA genes (V1-V2 regions)	There was no significant diversity or compositional differences between individuals taking or not antidepressants. Antidepressant users showed increased gamma-aminobutyric acid (GABA) III synthesis and GABA degradation.
(Vich Vila *et al.*, 2020)	Meta-analysis	N = 1124 General population (GP) N = 305 IBS N = 454 IBD F/M: 1120/763 Mean age: 44.8 (GP), 45.4 (IBS), 42.8 (IBD)	Bacterial taxonomic composition α-diversity (Shannon) β-diversity (Bray-Curtis) Inferred metabolic pathways (PICRUSt)	SSRIs (paroxetine, fluoxetine, sertraline, citalopram, escitalopram, fluvoxamine), TCAs (amitriptyline, maprotiline, clomipramine), other antidepressants (venlafaxine, duloxetine, mirtazapine, bupropion, trazodone)	Amplification and sequencing of 16S rRNA genes	The abundance of Streptococcus salivarius was increased in SSRI users. An increased abundance of Eubacterium ramulus was specific to participants using SSRIs. The use of TCAs was associated with an increased abundance of Clostridium leptum. The pathway involved in peptidoglycan maturation was decreased in the multi-drug meta-analysis of SSRI users compared to non-users.
(Vogt *et al.*, 2017)	Cross-sectional	Alzheimer’s disease N = 25 HCs N = 25 F/M: 35/15 Mean age: 71.3 (Alzheimer’s disease), 69.3 (HCs)	Bacterial taxonomic composition α-diversity (ACE, Chao1, Inverse Simpson, Shannon Index) β-diversity (Bray-Curtis, and weighted and unweighted UniFrac)	SSRIs	Amplification and sequencing of 16S rRNA genes (V4 regions)	There was no correlation between microbiota composition and diversity and antidepressant use.
(Wang *et al.*, 2023)	Longitudinal	MDD N = 110 HCs N = 166 F/M:167/109 Age range: 18–65	Bacterial taxonomic composition α-diversity (Chao1, Inverse Simpson, Shannon Index) β-diversity (Bray-Curtis) Inferred metabolic pathways Fecal and plasma metabolite profiling	Escitalopram 10–20 mg/day 12 weeks	Shotgun metagenomic sequencing	The treatment with escitalopram did not effectively shift the gut microbiome of MDD patients towards a healthier or more similar state to that of HCs.
(Ye *et al.*, 2021)	Longitudinal	MDD N = 26 HCs N = 28 F/M:42/12 Mean age: 26.04 (MDD), 26.04 (HCs)	Bacterial taxonomic composition α-diversity (Observed OTUs, Chao1, Shannon) β-diversity (unweighted UniFrac)	Vortioxetine 10 mg/day 4–8 weeks	Amplification and sequencing of 16S rRNA genes (V3-V4 regions)	Vortioxetine ameliorated depressive symptoms by promoting the reconstruction of the gut microbiota.
(Zhang *et al.*, 2019)	Pilot clinical trial	IBS + depression N = 6 Duloxetine-treated N = 9 Bifico-treated	Bacterial taxonomic composition α-diversity (Shannon) Serum cytokines Fecal short-chain fatty acids	Duloxetine 30–60 mg/day 8 weeks	Amplification and sequencing of 16S rRNA genes (V1-V3 regions)	The overall gut microbiota profile shifted after treatment with duloxetine. While the α-diversity of gut microbiota did not significantly change after treatment, the abundance of the genera Fecalibacterium, Lachnospiraceae incertae sedis, Escherichia/Shigella, and Sutterella showed a tendency to increase, and Erysipelotrichaceae incertae sedis tended to decrease.
(Zhernakova *et al.*, 2016)	Cross-sectional	General population N = 1135 F/M: 661/474 Mean age: 45.04 ± 13.60	Bacterial taxonomic composition α-diversity (Shannon, CoG richness, Gene richness) β-diversity (Bray-Curtis) Inferred metabolic pathways (PICRUSt)	TCAs, SSRIs, other antidepressants	Amplification and sequencing of 16S rRNA genes (V4 regions)	SSRIs show a positive association with Shannon’s diversity index, while other antidepressants showed a negative association. TCA use was significantly associated with an increase in two species from the genus Bacteroides and Coprococcus and a decrease in a species from the genus Eubacterium. There was a significant positive correlation of SSRIs with the lyxose degradation pathway and a negative correlation of other antidepressants with the methylerythritol phosphate pathway.

ACE, abundance-based coverage estimator; BD1, bipolar disorder type 1; CUMS, chronic unpredictable mild stress; dHDRS_24_, 24-item Hamilton Depression Rating Scale score change from baseline to endpoint; F/M, female/male ratio; HCs, healthy controls; IBD, inflammatory bowel disease; IBS, irritable bowel syndrome; MDD, major depressive disorder; NMDEA, N-(2-(7-methoxy-3,4-dihydroisoquinolin-1-yl)ethyl)acetamide hydrochloride; OTUs, Operational Taxonomic Units; PICRUSt, Phylogenetic Investigation of Communities by Reconstruction of Unobserved States; PCs, psychiatric controls; PPIs, proton pump inhibitors; PTSD, post-traumatic stress disorder; SNRIs; serotonin-norepinephrine reuptake inhibitors; SSRIs, selective serotonin reuptake inhibitors; TCAs, tricyclic antidepressants; TR, treatment-resistant.

### Commonly used techniques to study the gut microbiome

To aid the comprehension of the reviewed studies, it is useful to briefly outline the prevalent methods employed in gut microbiota characterization; however, discussing these technicalities more in detail goes beyond the scope of this review.

**Fig. 1:**
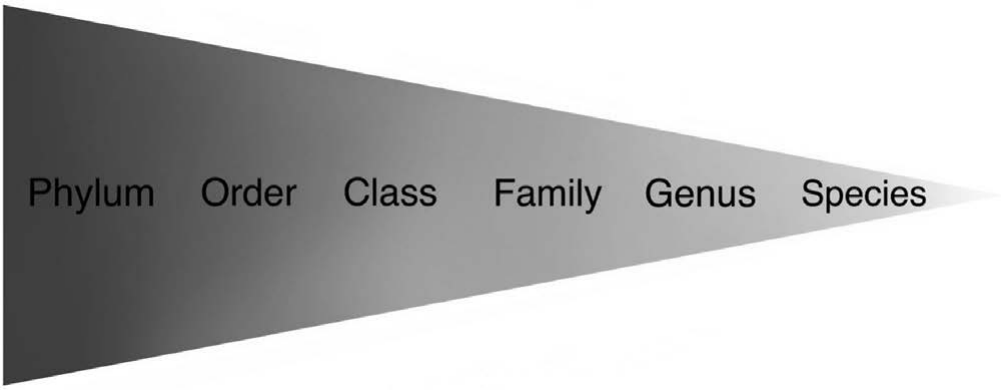
Hierarchical representation of bacterial taxonomic ranks in the gut microbiota.

Two sequencing techniques have significantly contributed to the understanding of the gut microbiome composition: the 16S prokaryotic ribosomal RNA (rRNA) gene sequencing and whole genome shotgun sequencing (Claesson *et al.*, 2017). In the majority of gut microbiome research, amplified sequences from the V3-V4 regions of the 16S rRNA gene serve as the basis for clustering into either Operational Taxonomic Units (OTUs) or, more recently, into higher-resolution Amplicon Sequence Variants (Claesson *et al.*, 2017). For a hierarchical representation of bacterial taxonomic ranks in the gut microbiota please refer to **Fig. 1**. Through bioinformatic analyses, the following standard metrics are also calculated to describe gut microbiota characteristics: (1) α-diversity, (2) β-diversity, and (3) the relative abundance of taxa (Knight *et al.*, 2018; Valles-Colomer *et al.*, 2019a). In particular, α-diversity measures the microbiota biodiversity within a sample through several diversity indices, the most widely used of which are the Shannon, Simpson, abundance-based coverage estimator (ACE), and Chao1 indices. β-diversity expresses the similarity or dissimilarity between (groups of) samples and it can be assessed by using quantitative metrics like the Bray–Curtis and weighted UniFrac, or qualitative metrics like the binary Jaccard and unweighted UniFrac (Hamady *et al.*, 2010).

The 16S rRNA gene sequencing technique does not provide direct information about the functional profile of bacterial communities. However, it is possible to infer which functions are likely to be associated with a marker gene using Phylogenetic Investigation of Communities by Reconstruction of Unobserved States (PICRUSt), an algorithm and software package that can predict functions encoded or carried out by the microbiome (Douglas *et al.*, 2018). PICRUSt maps known functional gene information onto the corresponding positions in the phylogenetic tree based on 16S rRNA sequences, inferring the presence of specific functional genes within the microbiome (Ijoma *et al.*, 2021). However, this method has several limitations. Currently, alternative techniques are more consistently employed to evaluate microbial function. One such approach, shotgun metagenomics, is the untargeted sequencing of all microbial genomes present in a sample. Shotgun genomics is particularly useful for sequencing complex genomes and is applied to characterize the taxonomic composition and functional capacity of microbial communities (Quince *et al.*, 2017).

## Results

A total of 50 studies matched the inclusion criteria and were included in the qualitative synthesis (the main characteristics of each study are summarized in Table 1).

### Antidepressant use and gut microbiota composition and diversity

#### Evidence from animal studies

Nine preclinical studies cross-sectionally investigated the association between antidepressants and gut microbiota composition. The samples were composed of healthy animals, stress-exposed animal models of depression, or other disease models. The antidepressants tested were buspirone, escitalopram, fluoxetine, ketamine, paroxetine, reboxetine, venlafaxine, and an analogue of agomelatine.

Healthy male rats were exposed to daily paroxetine or vehicle for 2 weeks and the gut microbiota was assessed 24 h after the end of the treatment or 1 month after withdrawal (Diviccaro *et al.*, 2022). Paroxetine did not influence α-diversity but affected β-diversity based on unweighted UniFrac distance after 24 h. In detail, there were significant alterations observed among paroxetine-treated rats in taxa belonging to the Firmicutes phylum, with an increase in the Enterococcaceae family, classified under the Bacilli class, and a decrease in the Clostridia class. Additionally, other less predominant phyla, such as Cyanobacteria, were also affected, showing a significant decrease in the Vampirivibrionia class (Diviccaro *et al.*, 2022). One month after paroxetine withdrawal, no difference in α- and β-diversity was observed between paroxetine-treated and control rats. However, a decrease in the Bifidobacteriaceae family within the Actinobacteria phylum, and Enterococcaceae and Lachnospiraceae family within the Firmicutes phylum was found in paroxetine-treated rats compared to controls. Conversely, an increase of taxa belonging to the Bacillota phylum and Staphylococcaceae family was observed in the treated rats (Diviccaro *et al.*, 2022). An earlier study examined the effects of low-dose ketamine on the microbial composition of stool samples from adult healthy Wistar rats. Ketamine exposure was associated with lower levels of opportunistic pathogens, such as Ruminococcus and Mucispirillum, and elevation of the beneficial genera Lactobacillus, Sarcina, and Turicibacter, as compared to saline-exposed controls (Getachew *et al.*, 2018). The microbial richness and diversity in animals treated with fluoxetine, escitalopram, and venlafaxine did not significantly differ from those of controls, which only received drinking water (Cussotto *et al.*, 2019). Only fluoxetine produced a decrease in the phylum Deferribacteres and the Prevotella and Succinivibrio genera (Cussotto *et al.*, 2019).

Inconsistently with these findings, another study investigating the effect of fluoxetine on rats exposed to stress during pregnancy and lactation detected differences in β-diversity between fluoxetine-treated rats and those treated with vehicle (Ramsteijn *et al.*, 2020). In particular, a significant increase in the abundance of the genera Prevotella and Ruminococcus – classified within the Bacteroidetes and Firmicutes phyla, respectively – and a decrease in the abundance of the Bacteroides genera, also belonging to the Bacteroidetes phylum, was found in rats undergoing antidepressant treatment (Ramsteijn *et al.*, 2020). Interestingly, in a study comparing healthy mice to others exposed to chronic unpredictable mild stress (CUMS) and treated with either fluoxetine or vehicle, fluoxetine directly attenuated the differences in microbial community composition between CUMS mice and healthy controls (Sun *et al.*, 2019). In particular, fluoxetine induced an increase in the Erysipelotrichia class, from the Firmicutes phylum, and Proteobacteria phylum compared to the CUMS group treated with vehicle, partially ameliorating the low bacterial diversity and attenuating the alteration of microbiota composition induced by CUMS (Sun *et al.*, 2019). A similar effect was observed in studies investigating the antidepressant buspirone (Kim *et al.*, 2021) and a dihydroquinoline analog of agomelatine [i.e., N-(2-(7-methoxy-3,4-dihydroisoquinolin-1-yl)ethyl) acetamide hydrochloride (NMDEA)] (An *et al.*, 2020). The former was found to partially reverse the altered gut microbiota of stressed mice by restoring the shifted β-diversity to that of control mice, while α-diversity was not influenced (Kim *et al.*, 2021). In particular, buspirone suppressed the Proteobacteria and elevated Bacteroidetes phylum populations (Kim *et al.*, 2021). The use of NMDEA on mice exposed to CUMS, which increased the bacterial community richness, resulted in a return to a normal level of richness (An *et al.*, 2020). Moreover, NMDEA was found to be able to correct stress-induced microbiota imbalance by reducing increased levels of Clostridia and increasing the abundance of probiotic bacteria, such as Lactobacillus (An *et al.*, 2020).

Antidepressants may also have detrimental effects on gut microbiota composition. A study assessing the effect of reboxetine on gut microbiota in diabetic compared to non-diabetic rats found differences in β-diversity between the control and diabetic groups. Additionally, there was a significant increase in specific taxa with pro-inflammatory capacity in both the diabetic and non-diabetic groups following reboxetine administration (Aydin *et al.*, 2021). Specifically, reboxetine treatment reduced Firmicutes and increased Bacteroides and Proteobacteria phyla, while increasing Prevotellaceae and decreasing Lactobacillaceae and Clostridiaceae families (Aydin *et al.*, 2021). Another study examined microbiota-related features in a naturally depressed mouse strain after 2 weeks of paroxetine treatment. The results showed that mice treated with paroxetine compared to vehicle-treated ones exhibited reduced gut microbiota α-diversity and higher elevated levels of primary and secondary bile acids - which are secreted directly by the liver or derive from bacterial metabolism in the colon, respectively. It is noteworthy that bile acids have been implicated in immune response and several gastrointestinal diseases (Dethloff *et al.*, 2020).

To summarize, one study found that antidepressants do not affect the gut microbiota composition of healthy rats (Cussotto *et al.*, 2019), while other studies demonstrated a significant influence of antidepressants on microbial community populations, in both healthy and pathological animals (Getachew *et al.*, 2018; Ramsteijn *et al.*, 2020; Aydin *et al.*, 2021; Diviccaro *et al.*, 2022). Three studies specifically indicated that antidepressants could reverse stress-induced microbiota imbalances, restoring it closer to the composition seen in control animals (Sun *et al.*, 2019; An *et al.*, 2020; Kim *et al.*, 2021). It should be noted, however, that two studies in healthy animals observed potential detrimental effects of antidepressant administration – specifically, a rise in taxa associated with inflammatory responses and a surge in bile acid levels, both of which have been implicated in gastrointestinal diseases (Dethloff *et al.*, 2020; Aydin *et al.*, 2021).

### Evidence from human studies

Eleven cross-sectional observational studies and a meta-analysis were conducted to investigate the association between antidepressant exposure and human gut microbiota composition. Most of the studies were conducted in population-based cohorts, examining the effect of various drugs, including antidepressants, on gut microbiota composition. Some studies considered samples of patients diagnosed with different conditions [i.e., Alzheimer’s disease, MDD, inflammatory bowel disease (IBD), irritable bowel syndrome (IBS)]. Antidepressants were considered indistinctly as a single group in some investigations, while others focused on different classes of antidepressants (SNRIs, SSRIs, and TCAs) or individual drugs (amitriptyline, bupropion, citalopram, clomipramine, duloxetine, escitalopram, fluoxetine, fluvoxamine, maprotiline, mirtazapine, paroxetine, sertraline, trazodone, and venlafaxine).

A population-based study found a significant correlation between the use of the SNRI venlafaxine and gut microbiome composition, with an increase in the Clostridium IV genus (Falony *et al.*, 2016). The use of TCAs was linked to an increase in two species from the genus Bacteroides and Coprococcus, and a decrease in a species from the genus Eubacterium (Zhernakova *et al.*, 2016). Another large observational study, examining the effects of 38 diseases and 51 medications on the gut microbiota, detected associations between SSRI use and lower abundance of many taxa, with the Turicibacteraceae family (belonging to the Firmicutes phylum) being the most affected (Jackson *et al.*, 2018). The effect of commonly used drugs, including antidepressants, on gut microbiota composition was investigated in a study including three independent Dutch cohorts. A significant association between antidepressant use and an increase of two taxa belonging to the Bacteroidetes phylum was found, but this association was lost after correction for proton-pomp inhibitors (PPIs) use (Imhann *et al.*, 2016). In a cohort including a high percentage of individuals with post-traumatic stress disorder, SSRIs and SNRIs were significantly correlated with changes in β-diversity, with an increase in the Ruminiclostridium (Firmicutes phylum) and Prevotella (Bacteroidetes phylum) genera. The SARI trazodone showed an inverse correlation with α-diversity (Stanislawski *et al.*, 2021), as well as a sample treated with SSRIs or SNRIs showed reduced α-diversity (Lin *et al.*, 2024). Another study conducted on elderly multimorbid patients found a positive association between antidepressants and several taxa, including Asteroleplasma, Helicobacter, Marinilactibacillus, unclassified members (UM) of the Bacilli class, and Succinivibrionacae family (Ticinesi *et al.*, 2017). The SSRI citalopram was also found to be significantly associated with an increased abundance of an OTU of the Enterobacteriaceae family (Rogers and Aronoff, 2016). The authors also reported that taking antidepressants plus non-steroidal anti-inflammatory drugs (NSAIDs) was linked to the increase of three species from the Bacteroidetes phylum and one from the Lachnospiracea family (Rogers and Aronoff, 2016).

On the other hand, three studies did not find any association between antidepressant prescription and gut microbiota composition. Two of these focused on specific age/disease groups, including 25 individuals with Alzheimer’s disease (Vogt *et al.*, 2017) and 160 adolescents (Thapa *et al.*, 2021), limiting the generalizability of the findings. Furthermore, they included small samples and showed potential confounders (Vogt *et al.*, 2017; Thapa *et al.*, 2021). A naturalistic study on 40 patients with depression and anxiety found no differences in diversity or composition between individuals taking antidepressants or not (Tomizawa *et al.*, 2021). It should be noted, however, that this study included a cohort on polytherapy with multiple psychotropic drugs.

A meta-analysis synthesized the evidence from a population-based cohort and two cohorts of patients with gastrointestinal diseases, such as IBD and IBS (Vich Vila *et al.*, 2020). An increase of Streptococcus salivarius and Eubacterium ramulus species (from the Bacillota and Firmicutes phyla, respectively) was seen in SSRI users, with the latter species remaining significant even after adjusting for other drug use. Likewise, the SSRIs (mainly paroxetine) were associated with an increase in Streptococcus salivarius when considering only patients with IBS. Finally, in the IBD cohort, TCA use was associated with an increased abundance of Clostridium leptum species (Firmicutes phylum) (Vich Vila *et al.*, 2020).

In line with results from preclinical investigations, most of the studies carried out in humans, further supported by a meta-analysis, demonstrated a significant association between the use of antidepressants and gut microbiota composition, although heterogeneous effects on different taxa were observed. This is likely due to, among others, differences in experimental design and laboratory techniques.

### The shift in gut microbiota profile after antidepressant treatment

#### Evidence from animal studies

Eight preclinical studies assessed longitudinal changes in gut microbiota composition and diversity using fecal samples collected at several time points before and after exposure to a specific antidepressant. The studies utilized either healthy animals or animal models of depression. The medications examined were amitriptyline, citalopram, fluoxetine, venlafaxine, and sertraline.

Three preclinical studies tested the effect of the SSRI fluoxetine on the gut microbiota of healthy mice. Analyzing stool samples collected from pregnant mice before and during an 8-day fluoxetine treatment, no effects were found, neither on the α and β-diversity of the maternal gut microbiota nor on the overall composition, with the exception of Lachnospiraceae COE1, which was the only taxon significantly and persistently increased (Vuong *et al.*, 2021). Likewise, following a 7-day administration of 10 mg/kg/day of fluoxetine, which elevates levels of intestinal serotonin [5-hydroxytryptamine (5-HT)], no significant difference in bacterial α-diversity was observed. However, there was a decrease in the abundance of the Turicibacter genus – a bacterium that directly imports 5-HT through a mechanism similar to mammalian serotonin transporter (SERT or 5-HTT) – and Clostridiaceae family (Fung *et al.*, 2019). On the other hand, a 29-day fluoxetine treatment regimen in healthy male mice led to time-dependent changes in gut microbial populations, specifically manifesting as a decrease in the abundance of the beneficial Lactobacillus johnsonii species (Lyte *et al.*, 2019). The authors also suggested that certain side effects of the medication may be attributable to these microbial alterations (Lyte *et al.*, 2019).

Five other studies focused on animal models of depression. In a model of corticosterone-induced stress, mice received oxytocin or the SSRI citalopram. The administration of oxytocin and corticosterone induced a similar shift in microbiota composition, while no changes were observed with citalopram (Dangoor *et al.*, 2021). Conversely, increased α-diversity was found after a 6-week treatment with fluoxetine and the TCA amitriptyline, in a CUMS-induced depression rat model. At the phylum level, the authors observed a decreased Firmicutes/Bacteroidetes ratio, due to enhanced Bacteroidetes and reduced Firmicutes relative abundance. This effect was mainly seen in the fluoxetine-treated arm. Following antidepressant treatment, at the genus level, there was an increase in the relative abundance of Bacteroides, Parabacteroides, Alistipes, and Butyricimonas. The latter are known as butyrate producers with anti-inflammatory properties. However, both fluoxetine and amitriptyline may also increase microbial subpopulations having a negative impact on health, such as Porphyromonadaceae family members and genus Alistipes (Zhang *et al.*, 2021). Venlafaxine also showed an effect but in the direction of restoring CUMS-induced alterations (Shen *et al.*, 2023). Additionally, a 2-week administration of sertraline in a mouse strain with natural depressive-like behavior led to still different results, showing no change in the overall microbial profile but a decrease in microbial α-diversity (McVey Neufeld *et al.*, 2019). Finally, fluoxetine did not restore CUMS induced alterations in another 4-week study but probably because of the low dose used (Dong *et al.*, 2023).

Overall, the longitudinal effect of antidepressants on gut microbiota exhibits significant variability, contingent upon factors such as the specific drug, treatment duration, and the animal model employed. While short-term fluoxetine treatments showed minimal impact on microbial diversity in healthy animals, long-term exposure led to changes in beneficial taxa. In animal models of depression, findings were inconsistent and heterogeneous, suggesting the influences of antidepressants on microbial populations with both detrimental and beneficial effects.

#### Evidence from human studies

Six studies and a meta-analysis longitudinally analyzed the association between antidepressant treatment and changes in gut microbiota composition in patients with MDD. The antidepressants administered included duloxetine, escitalopram, and vortioxetine, with some studies reporting the use of different molecules.

In a study involving 30 drug-naïve individuals with first-episode depression vs. 30 healthy controls, gut microbiota composition and α-diversity of patients were found to be different from healthy controls before treatment, but they became similar after escitalopram administration. In detail, the Firmicutes/Bacteroidetes ratio and the abundance of Lactobacillus significantly decreased, whilst the abundance of Christensenellaceae R-7 group, Eubacterium ruminantium group and Fusobacterium genus significantly increased. Also, β-diversity indices differed from baseline after treatment (Shen *et al.*, 2021). In a sample of 17 hospitalized patients with MDD, escitalopram treatment led to increased α-diversity, although with no differences in taxa abundance (Liśkiewicz *et al.*, 2019). However, a more recent study conducted by the same authors did not replicate the increase in α-diversity, possibly due to the use of different α-diversity measurement techniques (Liśkiewicz *et al.*, 2021). In a sample of 110 subjects with MDD, significant alterations following the treatment with escitalopram were observed in several species of spore-forming bacteria and the Firmicutes phylum (Wang *et al.*, 2023). Overall, escitalopram treatment was not effective in shifting the gut microbiome of the patients toward a state more similar to that of healthy subjects (Wang *et al.*, 2023). Similar to the first two mentioned studies using escitalopram, also vortioxetine was suggested to remodel gut microbiota based on data from 26 drug-naïve patients; however, in this case, no changes were found in α-diversity indices, but only in β-diversity (Ye *et al.*, 2021). At the phylum level, the abundance of Bacteroidetes and Proteobacteria gradually decreased, while the amount of Firmicutes gradually increased during vortioxetine treatment. At the genus level, the abundance of Alistipes and Prevotella decreased during treatment, while the abundance of Bifidobacterium - having beneficial effects on stress response and depression (e.g., (Yang *et al.*, 2017a)) -, Faecalibacterium, and Roseburia increased (Ye *et al.*, 2021). Lastly, in a pilot study, the overall profile of gut microbiota was analyzed in six patients with both IBS and depression before and after 8-week treatment with duloxetine. While the α-diversity of gut microbiota did not change after treatment, the abundance of Fecalibacterium, Lachnospiraceae incertae sedis, Escherichia/Shigella, and Sutterella showed a tendency to increase, while Erysipelotrichaceae incertae sedis tended to decrease (Zhang *et al.*, 2019).

A meta-analysis aimed to synthesize the evidence regarding the association between psychotropic use and changes in the gut microbiota (Minichino *et al.*, 2023). In particular, it revealed discrepancies among four studies regarding potential changes in taxonomic composition after antidepressant treatment. However, it consistently identified an increase in the abundance of Christensenellaceae, as evidenced in two distinct studies (Shen *et al.*, 2021; Ye *et al.*, 2021; Minichino *et al.*, 2023). Regarding diversity indices, pooled data on longitudinal studies showed significant differences in β-diversity (four studies) but not α-diversity (five studies) after antidepressant treatment (Minichino *et al.*, 2023).

Overall, longitudinal studies predominantly indicate that antidepressants like escitalopram and vortioxetine may remodel the gut microbiota in patients with MDD, aligning it more closely with that of healthy controls. Specifically, they influence α- and β-diversity, albeit inconsistently across studies and antidepressants, and alter the abundance of key microbial phyla such as Firmicutes and Bacteroidetes.

### Antidepressants and gut microbiota metabolic functions

#### Evidence from animal studies

Antidepressants have been shown to modulate not only neuronal function but also the metabolic activity of gut microbiota. Fung *et al.* (2019) provided pivotal evidence demonstrating that the gut bacterium Turicibacter sanguinis imports 5-HT via a mechanism reminiscent of the mammalian 5-HT transporter (SERT), and that fluoxetine may inhibit this process. In the presence of 5-HT, *Turicibacter sanguinis* reduces its expression of specific sporulation factors and membrane transporters, a modulation that is counteracted upon fluoxetine exposure. The inhibition of 5-HT uptake by fluoxetine plays a role in disrupting *Turicibacter sanguinis* competitive colonization within complex microbial communities (Fung *et al.*, 2019). Further emphasizing the relationship between antidepressants and microbial metabolic function, another study conducted in a CUMS rat model highlighted shifts in the gut microbiota metabolic pathways (Zhang *et al.*, 2021). Prior to antidepressant exposure, there was an upregulation in microbial functions related to membrane transport, carbohydrate metabolism, and signal transduction. However, a subsequent 6-week treatment with amitriptyline and fluoxetine resulted in a notable attenuation of these metabolic activities (Zhang *et al.*, 2021).

In summary, animal studies have shown that antidepressants, such as fluoxetine, modulate the metabolic activity of gut microbiota by inhibiting serotonin uptake mechanisms, affecting the colonization abilities of specific bacteria like Turicibacter sanguinis. Moreover, treatments with amitriptyline and fluoxetine were found to attenuate microbial functions related to membrane transport, carbohydrate metabolism, and signal transduction.

#### Evidence from human studies

The relationship between antidepressant use and gut microbiota functional profile has been investigated in five studies conducted on human samples and in a meta-analysis of three cohorts. Two studies focused on the effects of the SSRI escitalopram, while the remaining examined various classes of antidepressants, including SSRIs and TCAs. Two studies had a cross-sectional design and three longitudinally analyzed the change in gut microbiota metabolic functions.

In a Dutch population-based cohort, positive correlations between SSRI use and the lyxose degradation pathway, as well as a significant negative correlation between the use of other antidepressant classes and the methylerythritol phosphate pathway were found (Zhernakova *et al.*, 2016). In a meta-analysis testing 41 medication categories, SSRI users were found to have decreased peptidoglycan maturation pathway vs. non-users (Vich Vila *et al.*, 2020). Both these two studies considered samples with specific comorbidities (i.e., IBS).

Two studies provided evidence of a potential association between antidepressant use and GABAergic/glutamatergic metabolic pathways, known to be involved in MDD pathophysiology. In detail, antidepressant users showed increased GABA synthesis and GABA degradation, indicating that antidepressants may influence microbiome metabolism related to GABA (Tomizawa *et al.*, 2021).

Interestingly, even if antidepressants may induce a shift towards the gut microbial composition seen in healthy controls, there could be a tendency to return to the baseline condition after some time, mediated by the persistence of dysfunctions in microbiome metabolic function; this mechanism was suggested as possibly implicated in depression relapses (Shen *et al.*, 2021). In addition, microbiome metabolic pathways related to transport and catabolism, glycan biosynthesis and metabolism, cell motility, and membrane transport showed persistent alterations between patients and healthy controls, even after 4 weeks of treatment and a reduction in depressive symptomatology (Shen *et al.*, 2021). Consistently, escitalopram treatment demonstrated an inhibitory effect on microbial functions (Wang *et al.*, 2023).

In summary, the current evidence suggests that antidepressants may influence microbiota metabolic pathways, though the extent and clinical implications (e.g., possible connection with clinical benefits or drug side effects) of this modulation are not fully understood yet. Notably, antidepressant use has been linked with alterations in GABAergic and glutamatergic pathways, which are implicated in MDD pathophysiology. The metabolic profiles of the gut microbiome are observed to differ between individuals with MDD and healthy controls, and while antidepressants may temporarily shift these profiles toward a normative state, some discrepancies persist, potentially contributing to symptom relapse.

### Gut microbiota and antidepressant treatment outcomes

#### Evidence from animal studies

Seven preclinical studies investigated the association between the composition of the gut microbiota and the efficacy of antidepressants. The samples were composed of either healthy animals or animal models of depression. The majority focused on ketamine (or its metabolites) exposure; other antidepressants tested were desipramine, duloxetine, escitalopram, fluoxetine, and venlafaxine.

Four studies postulated that the antidepressant effect of ketamine may be partially mediated by changes in gut microbiota. A study investigated the efficacy of ketamine enantiomers on depressive-like symptoms in a rat social defeat stress model. (R)-ketamine exhibited greater efficacy compared to (S)-ketamine, and its administration led to a mitigation of the differences in gut microbiome composition between stressed mice and healthy controls. In detail, exposure of stressed rats to (R)-ketamine increased the levels bacteria from of the Mollicutes class and Butyricimonas genus, suggesting that the antidepressant effect of (R)-ketamine could potentially stem from these shifts in gut microbiome composition (Yang *et al.*, 2017b). Other studies considered the same or other animal models of depression and reported heterogeneous effects that ketamine enantiomers may have on different bacteria. In detail, a study found (R)-ketamine to be more efficacious than lanicemine – an experimental antidepressant with low-affinity, non-selective N-methyl-D-aspartate receptor antagonism activity. This superior effect was likely mediated by an increase in the levels of Bacteroidales and Clostridiales orders, as well as the Mogibacteriaceae family, coupled with a reduction of the Clostridium genus and Ruminococcaceae family (Qu *et al.*, 2017). In a lipopolysaccharide (LPS)-induced inflammation model of depression, the efficacy of ketamine treatment – measured by reduced immobility time in the forced swimming test (FST) – was significantly correlated with changes in specific bacterial taxa; these include an increase in the phylum Actinobacteria and its class Coriobacteriia, along with the order Clostridiales from the phylum Firmicutes, and the family Prevotellaceae and genus Alloprevotella from the phylum Bacteroidetes (Huang *et al.*, 2019). Further, a study focusing on norketamine (a major metabolite of ketamine) and the same depression model, found that (S)-norketamine but not I-norketamine exhibited antidepressant-like effects. Moreover, (S)-norketamine was particularly effective in attenuating LPS-induced elevations in specific bacteria like Bacterium ic1379, an as-yet-unclassified bacterial isolate, as well as Bacteroides sp. Marseille-P3166 and Bacteroides caecigallinarum, both species belonging to the genus Bacteroides of the phylum Bacteroidetes (Wang *et al.*, 2021).

In a naturally depressed mouse strain, the administration of five different antidepressants resulted in the decrease of the species Ruminococcus flavefaciens, belonging to the Firmicutes phylum (Lukić *et al.*, 2019). Noteworthy, in this study the supplementation with Ruminococcus flavefaciens was able to abolish the antidepressant effects of duloxetine, confirming that modifications in gut microbiota composition might be causally correlated with clinical benefits (Lukić *et al.*, 2019). Finally, in CUMS mice treated with escitalopram, the responder group was characterized by increased levels of the genus Prevotellaceae UCG-003, and depletion of the families Ruminococcaceae and Lactobacillaceae, which might therefore be biomarkers of antidepressant response (Duan *et al.*, 2021).

In summary, most preclinical studies focused on ketamine and described possible links between its antidepressant effects and changes in gut microbiota. Other antidepressants also demonstrate significant, yet complex, interactions with gut microbiome that affect their efficacy. Nevertheless, the identified associations with specific bacterial taxa were not consistently replicated across studies, therefore they necessitate further empirical validation.

#### Evidence from human studies

Twelve clinical studies and a meta-analysis investigated the association between gut microbiota features and antidepressant treatment outcomes. All of the studies considered exclusively individuals with depressive disorders, except one which included people with both depressive and anxiety disorders, and another which included a wider spectrum of psychiatric diagnoses in addition to depression. They all had a longitudinal design. The majority of studies examined individual classes of antidepressants, while others focused on specific molecules such as citalopram, escitalopram, levomilnacipran, paroxetine, and venlafaxine.

The most replicated finding was an association between response/remission to antidepressants and increased/higher α-diversity, at baseline (Minichino *et al.*, 2023; Wang *et al.*, 2023; Bharwani *et al*., 2020) and/or follow-up (Kurokawa *et al.*, 2021; Lee *et al.*, 2022; Wang *et al.*, 2023; Bharwani *et al*., 2020). However, not all studies were univocal, and one reported results in the opposite direction, that is, an increase in α-diversity in non-responders (Jiang *et al.*, 2015). One of the largest studies included 111 adult inpatients with various psychiatric diagnoses. Early in the course of hospitalization, bacterial richness and diversity were negatively associated with depression and anxiety severity at admission, and they were predictors of symptom remission from depression at discharge (Madan *et al.*, 2020). In contrast, another study observed no significant correlation between baseline α-diversity and MDD severity, both at admission and during treatment with escitalopram (Liśkiewicz *et al.*, 2021).

Our knowledge of the gut microbiome and its relevance to treatment outcomes has been enriched through comprehensive analyses focusing on taxonomic variations. Evidence has shown that the gut microbiota composition may differ depending on the individual response to antidepressant treatment, both in terms of baseline features or longitudinal changes (Jiang *et al*., 2015; Bharwani *et al*., 2020; Fontana *et al*., 2020; Madan *et al*., 2020; Kurokawa *et al*., 2021; Liśkiewicz *et al*., 2021; Dong *et al*., 2022; Lee *et al*., 2022). Notably, individuals in remission from depressive symptoms exhibited an enrichment at baseline in specific genera such as Faecalibacterium, Agathobacter, and Roseburia, all of which are constituents of the class Clostridia within the phylum Firmicutes (Lee *et al.*, 2022). These observations are corroborated by earlier research indicating that bacteria of the order Clostridiales were elevated only in remitters after 6 months of treatment compared to baseline (Bharwani *et al*., 2020). Furthermore, a negative correlation was found between the severity of depressive symptoms and the presence of these genera within the Clostridia class (Jiang *et al.*, 2015; Ye *et al.*, 2021). Regarding remission from depression at discharge, further compelling associations have been noted within the same class of Clostridia. Specifically, within the families Ruminococcaceae and Lachnospiraceae, distinct uncultured groups (UCGs) – like Ruminococcaceae UCG-002, UCG-010, UCG-014, and multiple Lachnospiraceae UCGs – have been found to have significant clinical implications (Madan *et al.*, 2020). Considering remission from anxiety, a unique yet partially overlapping microbiome signature was detected, predominantly within the Firmicutes phylum, specifically the Clostridiales order and the Ruminococcaceae family (Madan *et al.*, 2020). This pattern was also augmented by taxa from the Alcaligenaceae family, Burkholderiales order, and Betaproteobacteria class. Interestingly, the species Coprococcus catus was consistently associated with remission in both depressive and anxiety disorders (Madan *et al.*, 2020). Other authors found different baseline gut microbiota characteristics to be associated with treatment response. For example, individuals responsive to specific antidepressants such as citalopram, escitalopram, paroxetine, or venlafaxine showed reduced baseline abundances in the Actinobacteria phylum, particularly within the families Christensenellaceae and Eggerthellaceae (Dong *et al.*, 2022). This finding is also supported by genetic evidence. Indeed, in a Mendelian randomization study, Actinobacteria showed a protective causal effect on MDD (Chen *et al.*, 2022). Another study revealed that subjects who achieved remission after antidepressant treatment had significantly higher microbiome richness compared to the non-remitters group at both baseline and follow-up (Wang *et al.*, 2023). In addition, significant baseline differences in β-diversity of responders vs. non-responders were observed (Dong *et al.*, 2022). Another study in a sample with MDD found that the family Flavobacteriaceae, from the phylum Bacteroidetes, was uniquely present in individuals showing resistance to antidepressants. Additionally, these treatment-resistant individuals exclusively harbored genera such as Hungatella from the phylum Firmicutes, and Yersinia and Citrobacter from the phylum Proteobacteria, as well as the species Fenollaria timonensis. In contrast, treatment-responsive patients displayed an exclusive microbial signature that included entities from the phylum Elusimicrobia, as well as the genus Fenollaria and the species Robinsoniella sp. MCWD5, both of which belong to the phylum Firmicutes (Fontana *et al.*, 2020). A study with a similar design identified that relative abundance of Blautia, Coprococcus, and Bifidobacterium were predictive of SSRI response in MDD (Gao *et al.*, 2023).

Interestingly, three studies examined the relationship between microbiota metabolic pathways and antidepressant efficacy. Two studies found that altered bacterial functional profile was not predictive of treatment response in individuals with MDD (Liśkiewicz *et al.*, 2021; Tomizawa *et al.*, 2021). In contrast, in two additional studies, antidepressant non-responders exhibited increased inferred microbial glutamate synthesis compared to responders and remitters (Kurokawa *et al.*, 2021), and demonstrated greater susceptibility to treatment-induced perturbations in microbial function (Wang *et al.*, 2023).

In summary, studies on gut microbiota composition and antidepressant treatment outcomes generally reported significant relationships. Studies in human samples suggested that baseline gut microbiota composition could be linked to treatment outcomes, and α-diversity was implicated by several studies. Certain bacterial species, such as Faecalibacterium and Roseburia, both constituents of the Firmicutes phylum, are recurrently associated with remission and lower depression severity. However, results were often not consistent across studies, indicating the need for further research.

## Discussion

**Fig. 2:**
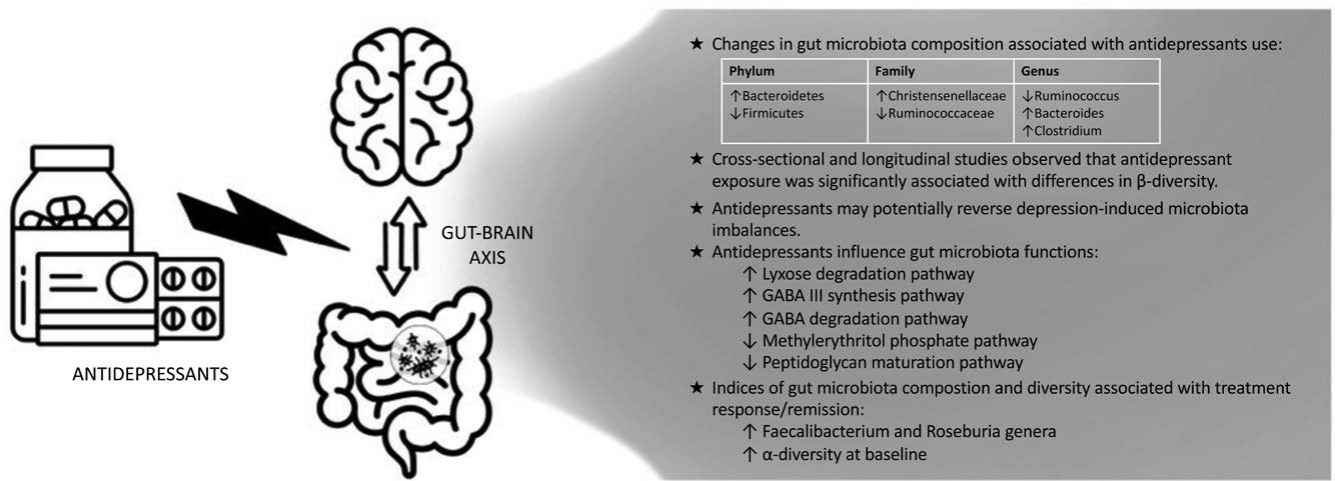
A graphical representation of the primary conclusions of this literature review. While these findings provide initial evidence, they should be interpreted with caution until further replication studies are conducted to verify their robustness and generalizability.

In this narrative review, we provide a comprehensive overview of the multifaceted relationship between gut microbiota and antidepressant treatment, shedding light on the current potential therapeutic implications for depression (**Fig. 2**). Antidepressants may alter both the diversity and composition of gut microbiota, as well as its metabolic pathways, attenuating the microbial differences observed between healthy controls and both stress-induced animals and patients with MDD. Specifically, antidepressant treatment is associated with an increase in the Bacteroidetes phylum, Christensenellaceae family, and Bacteroides and Clostridium genera, while decreasing the Firmicutes phylum, Ruminococcaceae family, and Ruminococcus genus. Antidepressant therapy also modulates gut microbiota function, with changes in lyxose degradation, peptidoglycan maturation, membrane transport, and methylerythritol phosphate pathways, as well as GABA metabolism and serotonin transport. Notably, metabolic function alterations may persist after cessation of antidepressant treatment, potentially elevating the risk of depression relapse. In terms of treatment responsiveness, emerging evidence suggests that increased baseline abundances of the genera Roseburia and Fecalibacterium, both constituents of the Firmicutes phylum, may serve as predictive markers of favorable treatment outcomes. Additionally, human studies consistently indicate that higher α-diversity correlates with better treatment outcomes, and preliminary evidence suggests a relationship between increased microbial glutamate synthesis and non-response.

More in detail, the standard metrics commonly used to describe gut microbiota composition (i.e., α-diversity, β-diversity, and relative abundance of taxa) were altered in relation to antidepressant exposure across most of the included studies. The β-diversity, which describes inter-individual differences in gut microbiota composition, was found to be the most consistently altered metric. Cross-sectional studies (Ramsteijn *et al.*, 2020; Kim *et al.*, 2021; Stanislawski *et al.*, 2021; Diviccaro *et al.*, 2022) observed that antidepressant exposure was significantly associated with differences in β-diversity. Significant dissimilarity in β-diversity was detected in longitudinal studies (Shen *et al.*, 2021; Ye *et al.*, 2021), before and after antidepressant administration, suggesting a possible causal relation.

Regarding the relative abundance of taxa, substantial evidence indicates an increase in the Bacteroidetes phylum, Christensenellaceae family, and Bacteroides and Clostridium genera, as well as a decrease in the Firmicutes phylum, Ruminococcaceae family, and Ruminococcus genus, in samples exposed to antidepressants. While there is conflicting evidence regarding the beneficial or detrimental effects of the Bacteroidetes phylum (Naseribafrouei *et al.*, 2014) and the Clostridium genus (Barandouzi *et al.*, 2020; Grenda *et al.*, 2022), the Bacteroides and Christensenellaceae families demonstrated anti-inflammatory properties and may play a positive role in several conditions. Specifically, Bacteroides species are considered promising candidates for next-generation probiotics (i.e., advanced types of beneficial bacteria or microbial formulations that are designed to provide enhanced health benefits beyond traditional probiotics (Barbosa *et al.*, 2022)), due to their ability to regulate the immune system, resulting in beneficial effects at multiple levels (Tan *et al.*, 2019). Christensenellaceae are emerging as important players in human health in different disease contexts, including obesity and IBD (Waters and Ley, 2019), and were found to be decreased in patients with affective disorders (Liu *et al.*, 2020; Coello *et al.*, 2021). On the other hand, the increased abundance of Ruminococcus genus and Ruminococcus species was associated with anhedonic and depressive-like symptoms in animal models (Davis *et al.*, 2017), even though these results were not replicated in human studies (Painold *et al.*, 2019; Maes *et al.*, 2023).

Antidepressant-induced alterations of the gut microbiome may be mediated by the antimicrobial potency of antidepressants, as demonstrated *in vitro*. SSRIs showed efficacy against Gram-positive bacteria and antifungal properties (Ayaz *et al.*, 2015; Silva *et al.*, 2017; Ait Chait *et al.*, 2020). They demonstrated antimicrobial properties at high concentrations and antimicrobial enhancer properties at lower concentrations, thereby synergistically reducing minimum inhibitory concentrations when combined with antibiotics (Li *et al.*, 2017). Consistent with this, a general upregulation of antimicrobial resistance genes in the gut microbiota was observed following escitalopram treatment (Wang *et al.*, 2023). TCAs showed anti-plasmid effects and the ability to hinder the growth of intestinal pathogens (Macedo *et al.*, 2017). Ketamine also exhibited antimicrobial activity against various pathobionts (Gocmen *et al.*, 2008; Begec *et al.*, 2013). Yet, specific underlying mechanisms remain unclear. The observed alterations in gut microbiota following antidepressant administration may partially explain gastrointestinal side effects (Oliva *et al.*, 2021), likely through the decrease in beneficial bacterial populations (Lyte *et al.*, 2019), or the increase in pro-inflammatory ones (Aydin *et al.*, 2021). Consistently, the antimicrobial potency of antibiotics was linked to mental health outcomes, possibly via their influence on gut microbial diversity (Dinan and Dinan, 2022).

Antidepressants have been shown to influence various metabolic pathways of the gut microbiome, reflecting their intricate relationship with the gut-brain axis. Antidepressant exposure was related to alterations in several pathways, including lyxose degradation (Zhernakova *et al.*, 2016), methylerythritol phosphate (Zhernakova *et al.*, 2016), peptidoglycan maturation (Vich Vila *et al.*, 2020), membrane transport (Shen *et al.*, 2021; Zhang *et al.*, 2021), as well as the GABAergic and the glutamatergic metabolic pathways (Kurokawa *et al.*, 2021; Tomizawa *et al.*, 2021). In line with these findings, peptidoglycan pathways were related to the antidepressant effect of physical exercise in a CUMS-induced depression model, and significant differences in membrane transport pathways were observed in depression-susceptible mice offspring compared to controls (Li *et al.*, 2023). There is also a known association between abnormal functioning of the glutamatergic system and depression (Mathews *et al.*, 2012), as well as between alterations in GABA signaling and both depression and anxiety (Valles-Colomer *et al.*, 2019b). The intricate connection between the serotonergic system and the gut microbiome becomes even more evident when examining the findings of Fung *et al.* (2019). They demonstrated the role of Turicibacter sanguinis, a gut bacterium, in both modulating and being modulated by 5-HT transport mechanisms. Interestingly, Turicibacter sanguinis imports 5-HT in a manner that is susceptible to inhibition by fluoxetine, a commonly prescribed SSRI (Fung *et al.*, 2019). This reciprocal modulation between 5-HT and Turicibacter sanguinis not only emphasizes the bidirectional communication between select gut bacteria, the host’s serotonergic system, and antidepressant therapy but also underscores the potential implications of such interactions on gut microbial ecology, particularly in the context of antidepressant interventions. Building on this connection, it is worth noting that both serotonin and indole-3-propionic acid (I3PA), a microbial-derived metabolite, originate from tryptophan. I3PA levels are significantly diminished in individuals with MDD and tend to increase after antidepressant treatment (Wang *et al.*, 2023). This suggests a potential indirect influence on the serotonergic pathway, given their shared precursor. Therefore, the current evidence suggests that antidepressants may also exert their effects by modulating metabolic pathways known to be involved in depression etiopathogenesis. Interestingly, the relation between antidepressants and metabolic pathways of the gut microbiome is bidirectional. Not only antidepressants may be able to alter the microbiome, but gut bacteria also possess enzymatic capabilities that can modify the bioavailability and effectiveness of these medications. The main mechanism implicated in this interplay is biotransformation, which refers to the microbial chemical modification of compounds (Koppel *et al.*, 2017). Recently, bacteria-drug interactions were shown to encompass bioaccumulation, which occurs when bacteria store compounds intracellularly without chemically modifying them. This phenomenon often does not affect significantly bacterial growth. Specifically, the bioaccumulation of duloxetine by several pathobionts was demonstrated, which resulted in reducing its bioavailability and potentially affecting its efficacy (Klünemann *et al.*, 2021). In addition, the effect of duloxetine was found to be abolished by the supplementation of Ruminococcus Flavefaciens, although the underlying mechanisms remain unknown (Lukić *et al.*, 2019).

Finally, a potential relationship was observed between microbiome composition and metabolomics and antidepressant treatment outcomes. Depression is a disease with a high risk of relapse and recurrence (Beshai *et al.*, 2011); evidence suggests that experiencing a relapse or recurrence of depression potentially heightens the risk of treatment resistance (Post, 1992). Identifying predictors of response and the most suitable treatment for each patient may reduce the burden of the disease and the related costs to society (Serretti, 2022a). Heightened α-diversity at baseline was the most implicated measure in predicting antidepressant treatment outcomes, and increased abundance of certain bacterial genera, such as Faecalibacterium and Roseburia, was associated with remission and lower depression severity (Fontana *et al.*, 2020; Duan *et al.*, 2021; Ye *et al.*, 2021; Lee *et al.*, 2022). Other studies reported that α-diversity increased during follow-up in responders (Kurokawa *et al.*, 2021; Lee *et al.*, 2022). This aligns with the evidence of an association between α-diversity and depression severity (Bosch *et al.*, 2022; Hu *et al.*, 2023).

Interestingly, specific alterations in taxa composition might be predictive of treatment resistance. For instance, bacteria belonging to the Proteobacteria phylum, such as Citrobacter and Yersinia, were found to be increased in patients with treatment-resistant depression (Fontana *et al.*, 2020). Administration of probiotics demonstrated promising efficacy in animal models of treatmentresistant depression (Palepu and Dandekar, 2022). Moreover, metagenomics analyses revealed that the presence of sporulation genes at baseline could predict the likelihood of achieving clinical remission following SSRI treatment. This raised the possibility of identifying baseline gut microbiota characteristics that could serve as predictors of antidepressant efficacy (Wang *et al.*, 2023).

The results of this review should be interpreted considering several limitations. Firstly, a standardized definition of the “normal microbiome” is lacking, both in terms of composition and metabolic functional profile. A primary reason for this is the high inter-individual variability in the human microbiome. Factors like age, sex, genetics, environment, physical exercise, dietary habits, comorbid diseases, and pharmacological treatments contribute significantly to differences in microbiome composition (Rothschild *et al.*, 2018; Pusceddu and Del Bas, 2020). Moreover, there is a duplicity in the role microorganisms may play in affecting physiological well-being, wherein the same microbe might possess the capacity to positively or negatively impact human health (Liu *et al.*, 2022a). Only a few of the included studies considered the multitude of variables that influence the human gut microbiota, notably lacking in-depth analysis of sex and age-specific variations. Such differences are particularly relevant considering the known interaction of sexual steroids on the gut microbiome (Pace and Watnick, 2021). For instance, only one study addressed the issue of elderly individuals with multimorbidity and polypharmacy (Ticinesi *et al.*, 2019). Secondly, although studying gut microbiota in mice has the advantage of providing standardized experimental conditions (Bastiaanssen *et al.*, 2020), the inconsistencies in results found for specific taxa and diversity indices among studies suggest a residual source of heterogeneity, such as the variety of antidepressants used (with fluoxetine being the most represented), the duration of drug exposure (from a single dose to multiple administrations over 6 weeks), the utilization of healthy mice or different models of disease, and whether the studies were conducted in wet or dry lab setting. Thirdly, there were also discrepancies in the results of studies conducted in human samples, both concerning diversity and taxonomic composition. These might be partially explained by differences in study design, drugs, treatment duration, sample size, and other characteristics of the sample. We noticed a substantial lack of randomized controlled trials (RCT), with only one pilot RCT available (Lee *et al.*, 2022). Several studies involved samples of 50 or fewer individuals or considered patients with diverse diagnoses and polypharmacotherapy. Confounding factors, with concurrent medication having the most significant impact, can lead to systematic errors in clinical research (Ilzarbe and Vieta, 2023). Diversity indices were not calculated uniformly across studies, and it is very challenging to compare the results. Of note, despite the long-standing assumption that increased bacterial diversity is beneficial for human health, it is now understood that diversity is likely neither favorable nor detrimental (Shade, 2017). Fourth, preclinical studies show limited comparability with the complexity of human pathophysiology. Therefore, the reproducibility of these findings in humans is largely uncertain (Liu *et al.*, 2022a).

In light of the discussed limitations, future studies should use standardized procedures, encompassing study designs, inclusion criteria, sequencing techniques, bacteria variability indices, and systematic consideration of confounders. This would enhance study replicability and the clinical relevance of findings. Special attention should be given to sex and age-specific variations, recognizing the complexity and significance of these factors in microbiome research. Some relevant research questions remain poorly investigated, such as the possible effect of antidepressants on bacteria response to other drugs, particularly antibiotics (Drew, 2023). It would be of interest to investigate the potential impact of other medications with off-target effects on the gut microbiome, such as NSAIDs (Maseda and Ricciotti, 2020), in modifying the response to antidepressants, and whether any synergistic effect can be exploited in certain patients groups. Another intriguing future possibility is to implement precision medicine strategies that take into account the individual gut microbiota profile, alongside other clinical and genetic factors, to predict individual treatment response or antidepressant tolerability (Fanelli *et al.*, 2022; Fusar-Poli *et al.*, 2022; Oliva *et al.*, 2022). In addition, well-designed clinical studies are needed to determine whether microbiota-targeting strategies could reverse depression-related alterations in the gut microbiome or be considered adjunctive therapy to standard antidepressants. Many strategies have been investigated in recent years, including dietary interventions, fecal microbiota transplantation (Kang *et al.*, 2017), probiotics (Liu *et al.*, 2023), prebiotics (Liu *et al.*, 2022b), or “psychobiotics” (i.e., bacteria with a potential mental health benefit) (Cussotto *et al.*, 2021). Preclinical findings were encouraging but only modest results were obtained in human samples.

## Conclusion

This review suggests that antidepressant use is associated with alterations in gut microbiota composition, with increasing evidence that antidepressant treatment may partially restore depression- and stress-induced alterations in microbiome composition and functionality. Gut microbiota has a potential role in modulating treatment response, and some microbiota characteristics may be correlated with treatment response. Further studies are needed to confirm these findings and elucidate the mechanisms underlying antidepressant-microbiota interactions. Future research should also explore the potential of microbiome-based interventions to complement traditional pharmacological approaches, and ultimately lead to more personalized, effective, and tolerated treatments for depression.

## Acknowledgements

We thank the support of The MNESYS – Mood and Psychosis Sub-Project (Spoke 5) Consortium (on behalf of the Consortium: Luigi Grassi (University of Ferrara, Ferrara, Italy), Alessio Maria Monteleone and Silvana Galderisi (University of Campania, Naples, Italy), Alessandro Bertolino (University of Bari, Bari, Italy), Pierluigi Politi (University of Pavia, Pavia, Italy), Mirella Ruggeri (University of Verona, Verona, Italy), Valdo Ricca (University of Florence, Florence, Italy), Luca Serafini (University of Genoa, Genoa, Italy), Cinzia Niolu (University Tor Vergata, Rome, Italy)). This work was supported by #NEXTGENERATIONEU (NGEU) and funded by the Italian Ministry of University and Research (MUR), National Recovery and Resilience Plan (NRRP), project MNESYS (PE0000006) – *A Multiscale integrated approach to the study of the nervous system in health and disease* (DN. 1553 11.10.2022).

### Conflicts of interest

C. Fabbri was a speaker for Janssen. A. Serretti is or has been a consultant/speaker for Abbott, Abbvie, Angelini, AstraZeneca, Clinical Data, Boehringer, Bristol-Myers Squibb, Eli Lilly, GlaxoSmithKline, Innovapharma, Italfarmaco, Janssen, Lundbeck, Naurex, Pfizer, Polifarma, Sanofi, Servier and Taliaz. E. Vieta has received grants and served as consultant, advisor, or CME speaker for the following entities: AB-Biotics, AbbVie, Angelini, Biogen, Biohaven, Boehringer-Ingelheim, Celon Pharma, Compass, Dainippon Sumitomo Pharma, Ethypharm, Ferrer, Gedeon Richter, GH Research, Glaxo-Smith Kline, Idorsia, Janssen, Lundbeck, Medincell, Novartis, Orion Corporation, Organon, Otsuka, Rovi, Sage, Sanofi-Aventis, Sunovion, Takeda, and Viatris, outside the submitted work. For the remaining authors, there are no conflicts of interest.

## Appendix. Search query (PubMed)

((microbiome[Title/Abstract] OR microbiota[Title/Abstract] OR bacteria[Title/Abstract] OR flora[Title/Abstract] OR microflora[Title/Abstract] OR dysbiosis[Title/Abstract])

**AND** (gut[Title/Abstract] OR gastroenteric[Title/Abstract] OR intestinal[Title/Abstract] OR enteric[Title/Abstract] OR bowel[Title/Abstract] OR oral[Title/Abstract] OR fecal[Title/Abstract])

**AND** (depression[Title/Abstract] OR depressive[Title/Abstract] OR MDD[Title/Abstract] OR antidepressant*[Title/Abstract] OR "norepinephrine reuptake inhibitors"[Title/Abstract] OR "serotonin–norepinephrine reuptake inhibitors"[Title/Abstract] OR "selective serotonin reuptake inhibitors"[Title/Abstract] OR "norepinephrine-dopamine reuptake inhibitors"[Title/Abstract] OR "monoamine oxidase inhibitors"[Title/Abstract] OR "serotonin antagonists and reuptake inhibitors"[Title/Abstract] OR TCA[Title/Abstract] OR MAOI[Title/Abstract] OR SSRI[Title/Abstract] OR SNRI[Title/Abstract] OR fluoxetine[Title/Abstract] OR fluvoxamine[Title/Abstract] OR escitalopram[Title/Abstract] OR citalopram[Title/Abstract] OR paroxetine[Title/Abstract] OR sertraline[Title/Abstract] OR paroxetine[Title/Abstract] OR venlafaxine[Title/Abstract] OR vortioxetine[Title/Abstract] OR duloxetine[Title/Abstract] OR mirtazapine[Title/Abstract] OR bupropion[Title/Abstract] OR trazodone[Title/Abstract] OR ketamine[Title/Abstract] OR nortriptyline[Title/Abstract] OR desvenlafaxine[Title/Abstract] OR amitriptyline[Title/Abstract] OR clomipramine[Title/Abstract])

**AND** (‘english’[Language]))
